# Effects of ECMF Isolated from Mining Areas on Water Status, Photosynthesis Capacity, and Lead Ion Transport of *Populus alba* Under Pb Stress

**DOI:** 10.3390/jof10120822

**Published:** 2024-11-26

**Authors:** Na Wu, Zhen Li, Fei Wu, Jing Tan

**Affiliations:** 1 Institute of Applied Biotechnology, College of Agronomy and Life Sciences, Shanxi Datong University, Datong 037009, China; zhenlingna90@126.com (N.W.); sqzr88@sina.com (J.T.); 2 College of Life Sciences, Northwest Normal University, Lanzhou 730070, China; wufei315@163.com

**Keywords:** ectomycorrhizal fungi, *Populus alba*, Pb stress, metallothionein gene expression, mining areas

## Abstract

Native ectomycorrhizal fungi (ECMF) are generally more effective than non-native ECMF in facilitating the phytoremediation of heavy metal (HM) ions from contaminated soils. This study aimed to investigate the contributions of four ECMF species—*Suillus luteus*, *Suillus flavidus*, *Suillus variegatus*, and *Gomphidius glutinosus*—that were isolated from mining areas to the growth, water status, photosynthesis, and metallothionein gene expression of *Populus alba* exposed to varying concentrations of lead (Pb). The experiment lasted two months and involved *P*. *alba* cuttings subjected to Pb concentrations of 0, 200, and 400 mg kg^−1^, representing no Pb stress, moderate Pb stress, and severe Pb stress, respectively. Results indicated that *S*. *flavidus* significantly enhanced the growth, water status, photosynthesis parameters, and metallothionein gene expression of *P*. *alba* under Pb stress, whereas *S*. *luteus* only exhibited positive effects under severe Pb stress. *S*. *variegatus* negatively affected the growth, water status, photosynthesis, and metallothionein gene expression of *P*. *alba* under Pb stress, while no significant difference was observed between the control treatment and *G*. *glutinosus* symbiosis. Therefore, *S*. *flavidus* and *S*. *luteus* are promising ECMF species for ecological restoration in mining areas, especially in *P*. *alba* woodlands.

## 1. Introduction

Heavy metal (HM) contamination is a significant environmental issue worldwide due to its toxic impact on ecological functions [1,2]. Although HMs are naturally occurring elements in soil, mining practices significantly increase their environmental presence [3,4]. Lead (Pb) is regarded as the most harmful HM due to its abundance and non-biodegradability [1,4,5]. An excessive Pb presence in soil adversely affects the morphological, physiological, and molecular functions of plants, despite being a non-essential element [6,7]. China, possessing the world’s second-largest Pb reserves, faces severe soil Pb contamination, which adversely impacts the construction of an ecological civilization [8]. Therefore, urgent remediation actions are necessary to address global soil HM contamination.

Poplar is an ideal model for plant biologists for exploring the molecular basis of tree responses to environmental stimuli owing to its sequenced genome [9]. *Populus alba* is widely cultivated in Shanxi Province, China—a typical mining area—as it serves as an important species for ecological restoration. Its features, including the ability to withstand environmental stresses, an extensive root system, and high water uptake, make it a suitable candidate for the phytoremediation of HM-polluted soils [9,10,11]. Moreover, metallothionein (MT) plays a crucial role in HM homeostasis in plants by binding metals [11,12,13]. MTs contain cysteine residues that facilitate metal binding, and the presence of multiple MT genes may reflect their diverse roles in heavy metal sequestration and other mechanisms essential for plant growth [11,13].

The influence of microorganisms on host plants has garnered significant attention due to its potential to mitigate the negative effects of HM stress on plants [4,14,15]. Mycorrhizal fungi can enhance the resistance, tolerance, and recovery of host plants under HM stress [16,17,18]. Previous research suggested that ECMF do not hinder HM absorption by host plants; rather, they promote it [18]. Some research proposed that mycorrhizal fungi could upregulate MT gene expression to enhance HM tolerance in host plants [9]. However, the physiological and molecular responses of plants to ECMF inoculation were highly variable and dependent on the fungal species [19].

Native ECMF generally provide more effective support for the phytoremediation of heavy metals (HM) from polluted soils than non-native ECMF [20,21]. In order to select potential and suitable application species for ecological restoration in mining areas, this study evaluated the contribution of four ECMF species (*Suillus luteus*, *S*. *flavidus*, *S*. *variegatus* and *Gomphidius glutinosus*), isolated from mining areas, to the growth, water status, photosynthesis, and MT gene expression of *P*. *alba* exposed to different Pb concentrations.

## 2. Materials and Methods

### 2.1. Plant and Soil Treatments

One-year-old *P*. *alba* plants were collected from a nursery at the Sanggan River Experimental Bureau in Datong, Shanxi Province, China. Cuttings were disinfected with 0.05% KMnO_4_ for 12 h and then rinsed three times with deionized water. The upper layer of soil, from a depth of 5 to 20 cm in the nursery poplar field, was filtered through a 2 mm sieve to provide a growth substrate for the cuttings. The sieved soil was then air-dried at 25 °C for 20 days, placed in a clean cloth bag, and sterilized in an autoclave at 121 °C and 0.11 MPa for 2 h to eliminate all microorganisms. The properties of the soil were as follows: pH (soil-to-water ratio: 1:5)—7.1; soil organic carbon—28.11 g kg^−1^; available nitrogen—17.36 mg kg^−1^; available phosphorus—16.89 mg kg^−1^; available potassium—39.17 g kg^−1^.

### 2.2. Fungal Preparation and Inoculation

ECMF inocula were stored in the microbiology laboratory of the College of Agronomy and Life Sciences at Shanxi Datong University. Four species, including *S*. *luteus* (SL), *S*. *flavidus* (SF), *S*. *variegatus* (SV) and *G*. *glutinosus* (GG), were isolated from the roots of plants in mining areas in Shanxi. The inocula of four ECMF species were prepared [22]. The fungi were initially cultivated on a potato dextrose agar (PDA) solid culture medium. After 2 weeks of growth, four circular blocks of medium (1 cm in diameter) were inoculated into 300 mL of potato dextrose liquid medium. The mixture of fungal mycelia was homogenized using a blender and then utilized as an inoculum.

### 2.3. Experimental Design

The experiment employed a completely randomized block design with two factors: ECMF inoculation status (inoculated sterile inoculum, SL, SF, SV and GG) and Pb stress gradients (0, 200, and 400 mg kg^−1^ Pb^2+^). Each treatment was replicated 20 times, resulting in a total of 300 pots (5 × 3 × 20 = 300). The cuttings were grown in a greenhouse at a temperature of 28 °C. The roots of *P*. *alba* were individually treated with 30 mL of a liquid medium containing SL, SF, SV, and GG. The remaining sixty cuttings in the non-inoculated treatments received 30 mL of autoclaved inoculum. After a maintenance period of 60 days, all cuttings were divided into three groups, and each group was watered with different concentrations of Pb^2+^. HM stress was maintained for an additional 60 days, after which the plants were harvested.

### 2.4. Ectomycorrhizal (ECM) Fungal Colonization Rate

At harvest, the fresh roots of six randomly selected plants were carefully washed with distilled water, bleached with 10% KOH at 90 °C until they became colorless, and then rinsed with distilled water three times, after which 10% H_2_O_2_ was added for 5 min to soften the samples. Following this, the fine roots were washed with distilled water three times, and 2% HCl was added for 5 min to cause acidification. We added with trypan blue dye (250 mL of lactic acid and glycerol, 0.5 g of trypan blue and 500 mL of water) to the washed fine roots and placed them in a water bath (90 °C) for 10 min. Then, the roots were subsequently decolorized with lactic acid–glycerol (1:1) at 90 °C for 20 min. The colonization was assessed via the grid line intersection method under an optical microscope [23].

### 2.5. Growth Status

To quantify the growth response, the height of the plants was measured as an indicator of the average growth achieved over the course of two months. This was determined from measurements at the beginning and end of the last 60-day Pb(NO_3_)_2_ treatment. Additionally, six samples, comprising both shoots and roots, were subjected to a temperature of 105 °C for 20 min within an oven to destroy enzyme activities, and they were then dried at 80 °C to a constant weight to determine the accumulation of biomass.

### 2.6. Relative Water Content (RWC) and Relative Electrolyte Leakage (REL)

At 10:00, after the total 120 days treatment, the fifth fully expanded leaf from the apex of the plant was collected from six randomly selected plants and weighed to determine the fresh weight (FW). Next, the leaves were placed in distilled water for 24 h to determine the turgid weight (TW). The leaves were then dried in a hot air oven at 70 °C until researching a constant weight to obtain the dry weight (DW). RWC was calculated as following: RWC (%) = (FW − DW) × 100%/(TW − DW).

20 leaf discs (1 cm^2^) from the fifth fully expanded leaf of the apex of the plants were placed in flasks containing 10 mL of deionized water. They were then vacuum-infiltrated for 30 min and maintained in water for 6 h. The electrical conductivity of the medium (EC1) was determined with a portable conductivity detector (LC116, Mettler Toledo Instruments Co., Ltd., Shanghai, China). The samples were boiled at 100 °C for 15 min to release all the electrolytes and then cooled to room temperature before the final electrical conductivity (EC2) was measured. The REL was calculated as follows: REL (%) = (EC1/EC2) × 100% [24].

### 2.7. Photosynthetic Capacity Parameters

Net photosynthesis rates (Pn), stomatal conductance (Gs), intercellular CO_2_ concentration (Ci), and transpiration rates (E) were determined for six mature leaves during a three-hour period using a Li-6400 portable photosynthesis system (Li-Cor Inc., Lincoln, NE, USA). The photosynthetic photon flux density was set to 1500 μmol m^−2^ s^−1^, the chamber CO_2_ concentration was set to 400 cm^3^ m^−3^, and the leaf temperature was set to 25 °C [25].

Before chlorophyll fluorescence measurements were performed, fully expanded leaves taken from six randomly selected cuttings from each treatment were placed in the dark for 30 min at 25 °C. The maximal fluorescence (Fm) and minimum fluorescence (Fo) yields were detected using a modulated chlorophyll fluorometer (Mini-Imaging-PAM, Walz, Germany). The maximum quantum yield (Fv/Fm) and actual quantum yield (ΦPSII) of photosystem II were calculated as follows: Fv/Fm = (Fm − Fo)/Fm; ΦPSII = (Fm′ − Fs)/Fm′. The photochemical quenching (qP) and nonphotochemical quenching (NPQ) were calculated as follows: qP = (Fs − Fo′)/(Fm′ − Fo′); NPQ = (Fm′ − Fo′)/(Fm − Fo) [26].

### 2.8. Pb Concentration and Translocation Factor

First, shoots and roots of cuttings were digested in Teflon vessels with concentrated HF and HNO_3_ acid solutions, and then the Pb concentrations in different plant organs were determined using flame atomic absorption spectrometry (FAAS, AA-7003A, Beijing, China). The translocation factor (TF) was estimated in order to depict the ability of the plants to accumulate HM [27], which is calculated as follows: TF = C_shoot_/C_root_. In this equation, C_shoot_ and C_root_ are the concentrations of Pb in the plant shoot and root.

### 2.9. Quantitative Real-Time PCR Analysis of MT Genes

Shoots and roots were used to extract RNA with an RNeasy Plant Kit (R6827-01 50T Omega, Norcross, GA, USA). RNA was detected using a Nanodrop 1000 spectrophotometer (Thermo Scientific NanoDrop, Waltham, MA, USA). First-strand cDNA was synthesized by a first-strand cDNA synthesis kit (Tiangen Biotech, Beijing, China). Transcripts of different treatments were determined using a CFX96 real-time PCR detection system (Bio-Rad, Hercules, CA, USA) and a Roche SYBR green system (Roche Diagnostics GmbH, SandhooferStraβe, Mannheim, Germany). The primers are listed in Table 1. A quantitative real-time polymerase chain reaction was conducted in a 20 μL reaction system, including 1 μL cDNA, 0.8 μL primer pairs (10 μM), 7.4 μL sterilized H_2_O, and 10 μL SYBR Premix Ex TaqTM II. The PCR amplification of PaMT1 was performed using the following program: we performed 3 min of denaturation at 95 °C and 45 cycles of 10 s for denaturation at 95 °C. We used 40 s for annealing at 60 °C, and 10 s for extension at 55 °C. The PCR amplifications of *PaMT2* and *PaMT3* were performed using the same program, except for annealing at 55 °C. As the endogenous control, the ubiquitin gene (UBQ) was selected to normalize the relative expression levels of MT genes [28]. Relative quantification was performed using the comparative 2^−ΔΔCT^ method [29].

### 2.10. Statistical Analysis

SPSS 26.0 software (SPSS Inc., Chicago, IL, USA) was used for statistical analysis. Two-way analysis of variance (ANOVA) was adapted to evaluate the significance of ECMF inoculation, Pb stress, and their interaction for cuttings at the significance level (*p* ≤ 0.05). The means were compared with Duncan’s multiple range test and the LSD test. Figures were made using Sigmaplot 15.0 (San Jose, CA, USA). For principal component analysis (PCA), all data above were standardized and subsequently computed. 

## 3. Results

### 3.1. ECM Fungal Colonization Rate

Upon harvest, no ECMF structures were observed in the roots of non-inoculated cuttings. In the inoculated treatments, typical ECMF structures were observed. The colonization rate of SL exceeded 30%, with significant differences observed between the control condition and severe Pb stress. The colonization intensity of SF exceeded 35%, with significant differences observed between the control condition and the Pb stress conditions. The colonization rate of GG was 25.39% under control conditions and 17.13% under severe Pb stress conditions. The colonization rates of SV were significantly lower than those of the other three species, and no significant differences were observed among different concentrations of Pb ions, indicating that poplar may not be a suitable host plant (Table 2).

### 3.2. Growth Status

**Figure 1:**
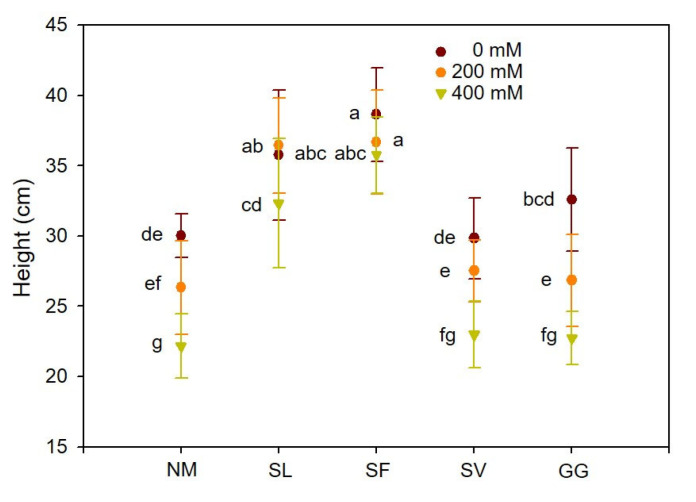
Effects of ECMF inoculation on height under different Pb treatments. NM, non-inoculation. Different letters indicate significant difference at *p* ≤ 0.05; the data are means ± SD (n = 6).

**Figure 2:**
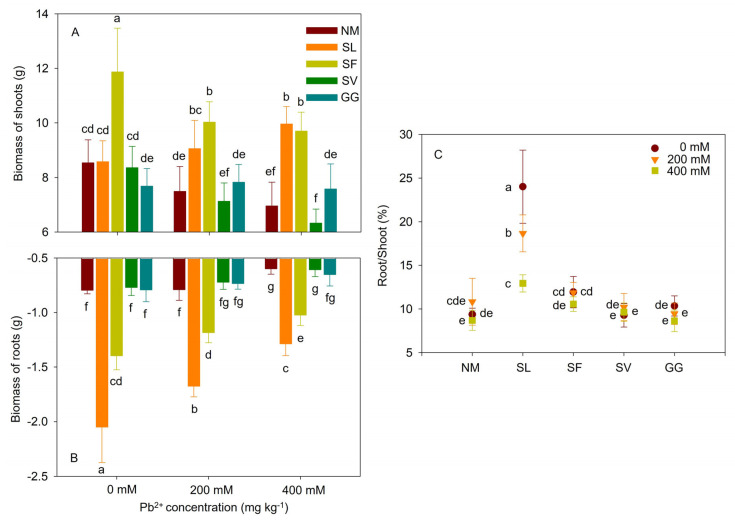
Effects of ECMF inoculation on biomass of shoots (**A**) and roots (**B**) accumulation and root/shoot ratio (**C**) under different Pb treatments. NM, non-inoculation. Different letters indicate significant difference at *p* ≤ 0.05; the data are means ± SD (n = 6).

Cuttings grown without Pb stress exhibited similar growth indices and biomass accumulation. Cuttings subjected to Pb stress showed declines in height and biomass. The four ECMF inoculations had distinct effects on the growth of cuttings. As shown in Figure 1, under moderate stress, the heights of cuttings inoculated with SL and SF were 38.37% and 39.33% higher than those of non-inoculated cuttings. Under severe stress, the heights of cuttings inoculated with SL and SF were 45.67% and 61.20% higher than those of non-inoculated cuttings. There was no significant effect on the height of cuttings inoculated with SV and GG at different lead concentrations. As shown in Figure 2, for SL, the biomasses of shoots and roots were 20.89% and 115.57% higher under moderate stress. The root/shoot ratio was significantly increased by SL under moderate stress. Furthermore, mycorrhizal cuttings were 43.14% and 115.06% greater than those of non-inoculated cuttings under severe stress. In the case of SF symbiosis, the biomasses of shoots and roots were 33.90% and 49.68% higher than those of non-inoculated cuttings under moderate stress, and 39.40% and 71.04% higher than those under severe stress. However, no significant effects of SV and GG inoculations on the biomass of shoots and roots were observed for different lead concentrations. Therefore, compared to SV and GG inoculations, cuttings with SL and SF symbiosis exhibited significantly greater height and biomass values under Pb stress. Additionally, SL symbiosis significantly increased the root/shoot ratio, raising it by 72.29% under moderate Pb stress. One-way ANOVA indicated that the height and biomass values of shoots and roots were significantly affected by Pb stress and ECMF inoculation. Two-way ANOVA indicated that the biomass values of shoots and roots were significantly affected by the interaction between Pb^2+^ treatment and ECMF inoculation (Table 3).

### 3.3. Relative Water Content (RWC) and Relative Electrolyte Leakage (REL)

**Figure 3:**
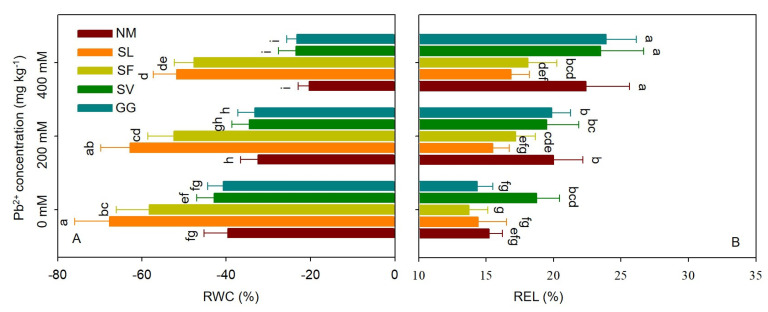
Effects of ECMF inoculation on RWC (**A**) and REL (**B**) under different Pb treatments. NM, non-inoculation. Different letters indicate significant difference at *p* ≤ 0.05; the data are means ± SD (n = 6).

Pb stress negatively affected the relative water content (RWC), counteracting the effects of SL and SF inoculation (Figure 3A). The RWC values in the leaves of cuttings inoculated with SL symbiosis were 93.38% and 61.22% higher under moderate stress compared to cuttings not subjected to Pb stress. The RWC values in the leaves of cuttings inoculated with SF symbiosis were 154.49% and 134.25% higher under moderate stress compared to cuttings not subjected to Pb stress. Pb stress significantly increased relative electrolyte leakage (REL) in leaves, while inoculation with SL and SF significantly decreased REL (Figure 3B). For SL, the REL values in leaves under moderate and severe Pb stress were 22.51% and 24.89% lower, respectively, than those observed in non-Pb stress cuttings. For SF, the REL values in leaves under moderate and severe Pb stress were 14.06% and 19.24% lower, respectively, than those observed in non-Pb stress cuttings. One-way ANOVA indicated that RWC and REL in leaves were significantly affected by Pb stress and ECMF species. Two-way ANOVA indicated that the interaction between Pb treatment and ECMF inoculation significantly affected REL in leaves (Table 3).

### 3.4. Gas Exchange Parameters

**Figure 4:**
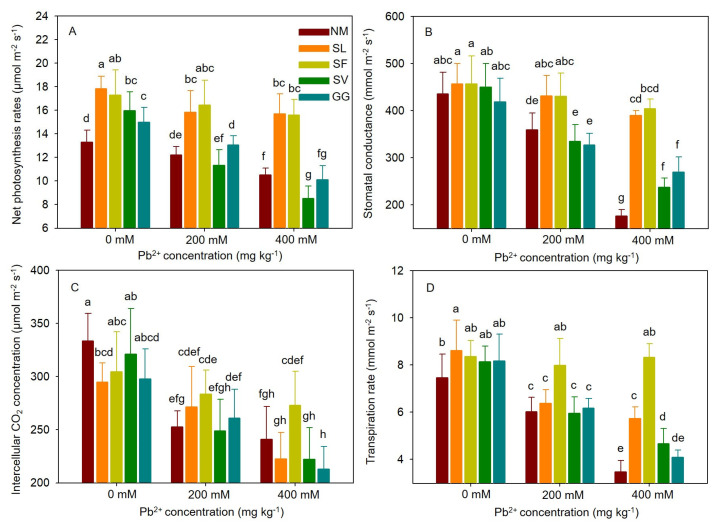
Effects of ECMF inoculation on gas exchange parameters ((**A**), net photosynthesis; (**B**), stomatal conductance; (**C**), intercellular CO_2_ concentration; (**D**), transpiration rates.) under different Pb treatments. NM, non-inoculation. Different letters indicate significant difference at *p* ≤ 0.05; the data are means ± SD (n = 6).

Under Pb stress, cuttings exhibited lower net photosynthesis (Pn), stomatal conductance (Gs), intercellular CO_2_ concentration (Ci), and transpiration rates (E) compared to cuttings not subjected to Pb stress. Inoculation with SL and SF positively affected the gas exchange parameters in cuttings, particularly under Pb stress (Figure 4). For SL, inoculated cuttings exhibited higher Pn (29.53%), Gs (19.98%), Ci (7.48%), and E (6.13%) values under moderate stress compared to non-inoculated cuttings. Under severe stress, inoculated cuttings showed higher Pn (49.43%), Gs (120.50%), and E (65.21%) values, but lower Ci (7.64%) values. For SF, inoculated cuttings exhibited higher Pn (34.55%), Gs (19.74%), Ci (12.17%), and E (32.90%) values under moderate stress compared to non-inoculated cuttings. Under severe stress, inoculated cuttings showed higher Pn (48.35%), Gs (128.57%), Ci (13.26%), and E (139.92%). No significant effects on gas exchange parameters were observed after inoculation with SV and GG under moderate stress. One-way ANOVA indicated that gas exchange parameters were significantly affected by Pb stress and ECMF species. Two-way ANOVA indicated that the interaction between Pb treatment and ECMF inoculation significantly affected gas exchange parameters (Table 3).

### 3.5. Chlorophyll Fluorescence Parameters

**Figure 5:**
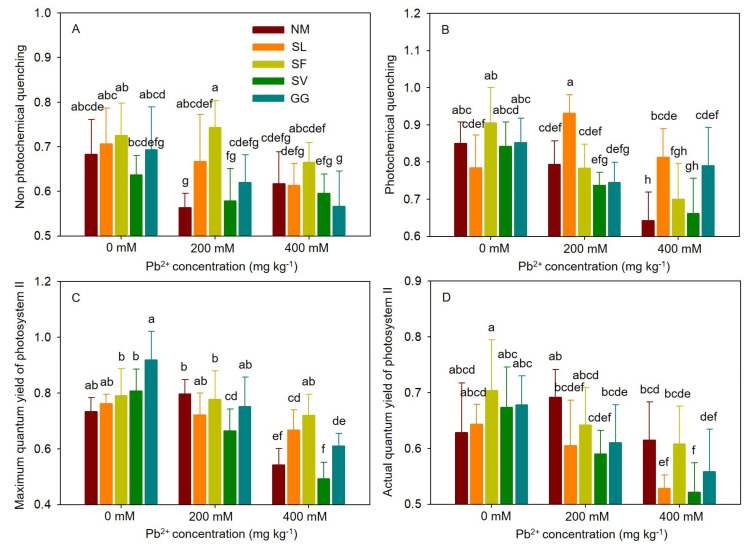
Effects of ECMF inoculation on chlorophyll fluorescence parameters ((**A**), nonphotochemical quenching; (**B**), photochemical quenching; (**C**), maximum quantum yield of photosystem II; (**D**), actual quantum yield of photosystem II) under different Pb treatments. NM, non-inoculation. Different letters indicate significant difference at *p* ≤ 0.05; the data are means ± SD (n = 6).

As shown in Figure 5, non-inoculated cuttings held in moderately contaminated soil exhibited 17.56% lower nonphotochemical quenching (NPQ) compared to those in non-contaminated soil (Figure 5). In severely contaminated soil, non-inoculated cuttings showed 24.51% lower photochemical quenching (qP) and 26.13% lower maximum quantum yield (Fv/Fm) compared to those in non-contaminated conditions. Compared to non-inoculated cuttings, SL inoculation resulted in higher qP (17.45%) and NPQ (18.36%) values under moderate stress. Under severe Pb stress, it also resulted in higher qP (26.74%) values and a higher maximum quantum yield of photosystem II (Fv/Fm) (23.08%). Compared to non-inoculated cuttings, SF inoculation resulted in significantly higher NPQ (31.96%) under moderate Pb stress and a significantly higher Fv/Fm (32.92%) under severe heavy metal stress. Inoculation with SV significantly decreased Fv/Fm and the actual quantum yield of photosystem II (ΦPSII) by 16.74% and 14.70%, respectively, at a concentration of 200 mM Pb. Following SV inoculation at a concentration of 400 mM Pb, mycorrhizal cuttings exhibited a lower ΦPSII (15.17%) compared to non-mycorrhizal cuttings. Inoculation with GG significantly increased qP by 23.89% at a concentration of 400 mM Pb. No significant effects on other chlorophyll fluorescence parameters were observed following GG inoculation under Pb stress. One-way ANOVA indicated that chlorophyll fluorescence parameters were significantly affected by Pb stress and ECMF species. Two-way ANOVA indicated that qP and ΦPSII were significantly affected by the interaction between Pb treatment and ECMF inoculation (Table 3).

### 3.6. Pb Accumulation and Translocation Factor

**Figure 6:**
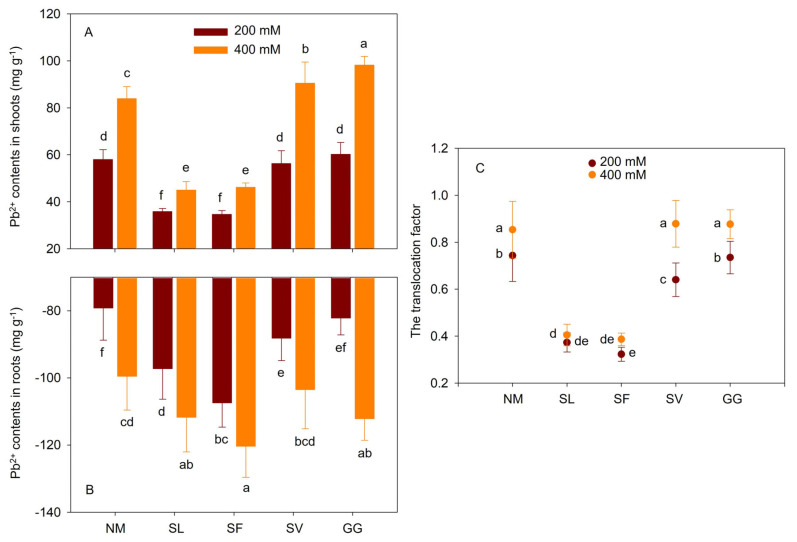
Effects of ECMF inoculation on Pb contents in shoots (**A**) and roots (**B**), and translocation factor (**C**) under different Pb treatments. NM, non-inoculation. Different letters indicate significant difference at *p* ≤ 0.05; the data are means ± SD (n = 6).

Pb stress increased the Pb content in the shoots and roots of the plants (Figure 6). When 200 mM Pb(NO_3_)_2_ was applied, inoculation with SL and SF significantly decreased Pb^2+^ levels in shoots. These fell by 38.26% and 40.27%, respectively, compared to non-mycorrhizal cuttings. However, SL and SF inoculation significantly increased Pb levels in shoots and roots. These rose by 22.82% and 35.73%, respectively, compared to non-mycorrhizal cuttings. When 400 mM Pb(NO_3_)_2_ was applied, inoculation with SL and SF significantly decreased Pb^2+^ levels in shoots. These fell by 46.40% and 44.99%, respectively, compared to non-mycorrhizal cuttings. Conversely, SL and SF inoculation significantly increased Pb levels in shoots and roots. These rose by 12.23% and 20.92%, respectively, compared to non-mycorrhizal cuttings.

No significant effects of SV and GG inoculations on Pb content in shoots were observed under moderate Pb stress conditions. Additionally, no significant effects were observed on Pb content from SV inoculation under severe Pb stress conditions or from GG inoculation in roots under moderate stress conditions. However, cuttings inoculated with SV and GG exhibited higher Pb^2+^ content in shoots, increasing by 7.78% and 17.03% under severe Pb stress, respectively. Following SV inoculation under moderate Pb stress, mycorrhizal cuttings exhibited 11.37% higher Pb content in roots compared to non-mycorrhizal cuttings. Following GG inoculation under severe Pb stress, mycorrhizal cuttings exhibited 12.67% higher Pb content in roots compared to non-mycorrhizal cuttings. Two-way ANOVA indicated that the Pb content in the shoots and roots was significantly affected by the interaction between ECMF species and Pb treatment (Table 3). Pb stress increased the translocation factor. When 200 mM Pb(NO_3_)_2_ was applied, inoculation with SL and SF significantly decreased the translocation factor by 49.78% and 56.51%, respectively, compared to non-mycorrhizal cuttings. When 400 mM Pb(NO_3_)_2_ was applied, inoculation with SL and SF significantly decreased the translocation factor by 52.54% and 54.68%, respectively, compared to non-mycorrhizal cuttings (Figure 6). One-way ANOVA indicated that the translocation factor was significantly affected by Pb stress and ECMF species. Two-way ANOVA indicated that the translocation factor was significantly affected by the interaction between ECMF species and Pb^2+^ treatment (Table 3).

### 3.7. The Relative Expression of MT Genes

**Figure 7:**
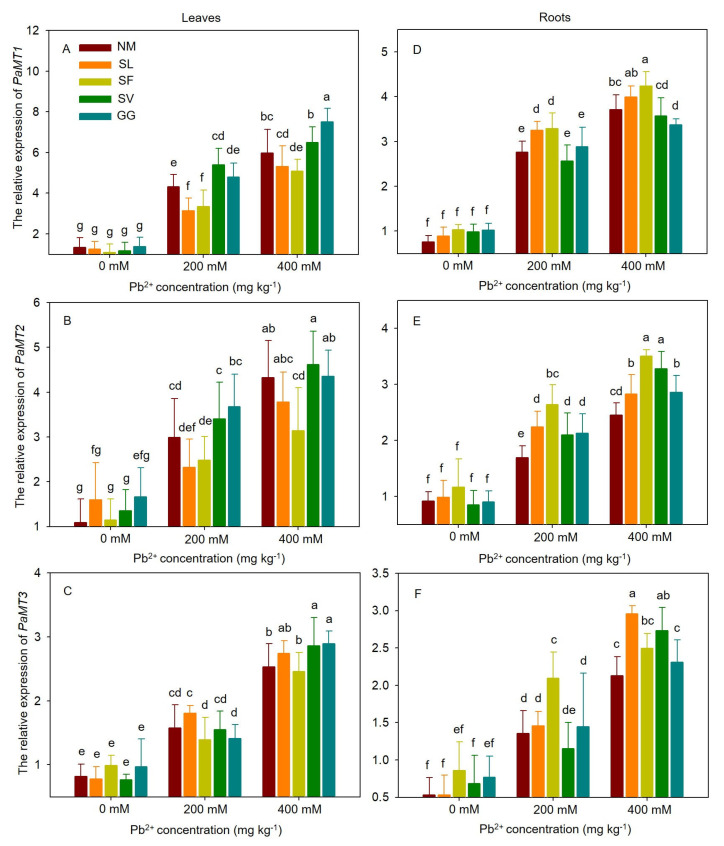
Effects of ECMF inoculation on *PaMT*s ((**A**), *PaMT1* in leaves; (**B**), *PaMT2* in leaves; (**C**), *PaMT3* in leaves; (**D**), *PaMT1* in roots; (**E**), *PaMT2* in roots; (**F**), *PaMT3* in roots) expression under different Pb treatments. NM, non-inoculation. Different letters indicate significant difference at *p* ≤ 0.05; the data are means ± SD (n = 6).

This study aimed to investigate the effect of ECMF colonization. The results of quantitative real-time PCR analyses, which evaluated steady-state transcript levels of *PaMT* genes, are presented by comparing relative expression levels in mycorrhizal cuttings and non-mycorrhizal plants under Pb stress (Figure 7). Regardless of ECMF inoculation status, HM stress upregulated the relative expression levels of *PaMT1*, *PaMT2*, and *PaMT3*. The relative expression of *PaMT1* increased rapidly in both leaves and roots. Inoculation with SL and SF significantly decreased the relative expression of *PaMT1* in leaves compared to the control conditions, falling by 27.49% and 22.45% under moderate Pb stress and by 11.03% and 14.90% under severe Pb stress. Cuttings inoculated with SV exhibited a 25.02% increase in *PaMT1* expression in leaves under moderate Pb stress, while those inoculated with GG showed a 25.56% increase under severe Pb stress. The expression of *PaMT1* in roots increased 1.17-fold and 1.19-fold due to SL and SF inoculation under moderate Pb stress compared to the control. Under severe Pb stress, compared to the control, the expression of *PaMT1* in roots increased 1.14-fold due to SF inoculation and decreased 0.91-fold due to GG inoculation. The symbiosis of the four ECMF species had no significant effect on the relative expression of *PaMT2* in leaves under moderate stress conditions. When 400 mM Pb(NO_3_)_2_ was applied, the expression levels of *PaMT2* in leaves with SF inoculation were 27.35% lower than those in non-inoculated cuttings. When 200 mM Pb(NO_3_)_2_ was applied, the relative expression levels of *PaMT2* in roots with SL, SF, SV, and GG inoculations were 32.02%, 55.89%, 23.77%, and 25.44% higher than those in non-inoculated cuttings. When 400 mM Pb(NO_3_)_2_ was applied, the relative expression levels of *PaMT2* in roots with SL, SF, SV, and GG inoculations were 15.38%, 43.06%, 33.88%, and 16.80% higher than those in non-inoculated cuttings. Compared to the control, the relative expression of *PaMT3* in leaves was upregulated 1.13-fold and 1.14-fold with SV and GG inoculations, respectively, under 400 mM Pb(NO_3_)_2_. When 200 mM Pb(NO_3_)_2_ was applied, the expression levels of *PaMT3* in roots with SF inoculation were 54.42% higher than those in non-inoculated cuttings. The relative expression of *PaMT3* in roots was upregulated 1.39-fold and 1.29-fold with SL and SV inoculations, respectively, under 400 mM Pb(NO_3_)_2_ treatment compared to the control. One-way ANOVA indicated that the relative expression of MT genes was significantly affected by Pb stress and ECMF species, except for *PaMT3* expression in leaves. Two-way ANOVA showed that except, for *PaMT2* expression in leaves, the relative expression of the other MT genes was significantly affected by the interaction between Pb treatment and ECMF inoculation (Table 3).

### 3.8. PCA Analysis

**Figure 8:**
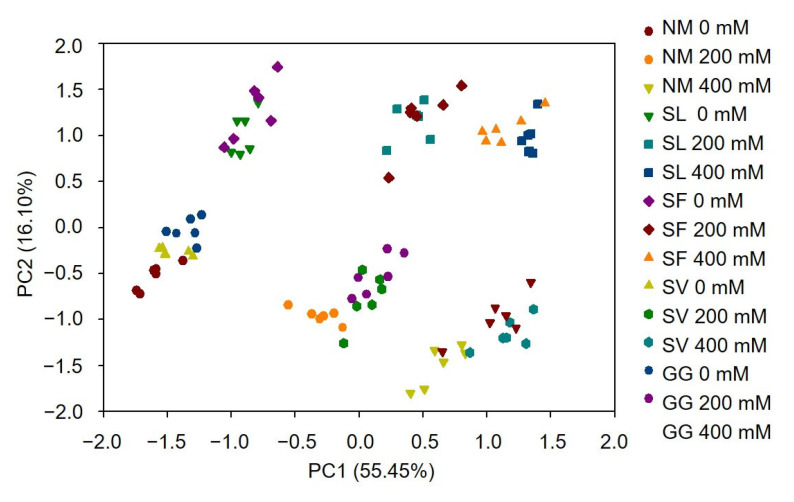
PCA results. NM, non-inoculation.

To investigate the physiological and molecular response patterns to Pb stress and ECM formation, PCA was conducted using experimental data sets. All parameters were reduced to the fewest and most representative dimensions, allowing PCA to convert large data sets into representative scores for each sample. These scores were used to create a PCA figure, which can be interpreted as a measure of distance between the samples and treatments (Figure 8). Principal component 1 (PC1) isolated the effect of HM stress, while principal component 2 (PC2) clearly separated the variation seen due to ECMF inoculation. PC1 and PC2 accounted for 55.45% and 16.10% of the total variation, respectively. The relative expression of metallothionein genes, Pb^2+^ content, translocation factor, and REL were key contributors to PC1, while colonization rates, growth status, photosynthesis indices, and RWC were key contributors to PC2. Furthermore, the distance between samples with SL and SF inoculations was closer than that among samples with non-inoculated, SV, and GG inoculations. These PCA results indicate that the four ECMF species exhibit distinct responses to Pb stress, and that SL and SF inoculations positively impact the heavy metal tolerance of *P*. *alba*.

## 4. Discussion

Heavy metal (HM) stress adversely affects plants at both physiological and molecular levels, significantly limiting their growth and biomass accumulation [1,4,30]. However, the use of ECMF species in phytoremediation has proven effective in restoring HM-contaminated soils [31,32,33]. Our results indicated that Pb stress significantly inhibited the growth of poplar cuttings; however, two specific ECMF species effectively alleviated these negative effects. Two mechanisms have been proposed to explain the reduced damage in mycorrhizal cuttings: enhanced photosynthetic capacity and the reduced accumulation of lead ions in shoots [30,34]. Consistent with this, our experimental findings demonstrated that inoculation with SL and SF improved the growth of host plants under Pb stress by enhancing photosynthesis and inhibiting the transport of Pb^2+^ from roots to shoots. In contrast, inoculation with SV and GG did not mitigate the damage caused by Pb stress. *P*. *alba* exhibited a higher ECMF colonization rate with SL and SF compared to SV and GG, indicating that poplar is a suitable host for these ECMF species.

Plant growth is a prominent indicator of development in response to ECMF inoculation and abiotic stress [23,35,36]. In our study, despite the inhibitory effects of Pb stress on cutting growth and biomass, cuttings inoculated with SL and SF exhibited better growth than non-mycorrhizal cuttings under Pb stress. This finding suggests that these two ECMF species can mitigate the adverse effects of Pb stress in poplar trees. Furthermore, the beneficial effects of ECMF inoculation on growth and biomass accumulation under HM stress have been observed in other plant species, including *Populus* × *canescens* [31], *Pinus sylvestris* [33], and *Pinus halepensis* [37].

HM stress can inhibit water uptake in plants, leading to reduced growth, as water uptake is essential for metabolic functions [1,4,7]. Our study found that HM stress decreased the RWC, especially in non-inoculated treatments, confirming previous research [38,39,40]. Furthermore, inoculating SL and SF had a more pronounced effect in terms of improving RWC under HM conditions. This suggests that these ECMF species enhance the self-protection ability of host plants by increasing water uptake under HM stress, thereby maintaining sufficient water availability to cope with stress [39].

Another important factor in assessing membrane damage in plants under HM stress is REL [41]. Lower REL values indicate minimal membrane injury and higher stress tolerance [42]. Our study found that HM stress significantly increased REL in *P*. *alba*, consistent with previous findings [41]. However, inoculating SL and SF significantly decreased REL in poplar cuttings, indicating that these ECMF species can maintain low REL levels in leaves, thereby enabling the cuttings to withstand Pb stress better.

Photosynthetic capacity is essential for plants to acquire energy and is one of the most sensitive processes affected by HM stress [6,42,43]. Numerous studies have shown that lead stress adversely affects the photosynthetic capacity of plants, leading to reduced photosynthesis [44,45], which aligns with our findings. Mycorrhizal associations can increase the absorptive surface area of plants by extending fungal hyphae beyond the root hair zone into the rhizosphere [34]. Inoculation with SF under moderate and severe heavy metal stress significantly increased the net photosynthesis rates, stomatal conductance, and transpiration rates of poplar cuttings. This improvement may correlate with enhanced water status and reduced lead accumulation in leaves under HM stress.

Chlorophyll fluorescence parameters are powerful tools for elucidating the function of the photosynthetic apparatus and are widely used in physiological studies [46,47]. The negative impact of heavy metals on photochemical quenching may reflect the increased closure of reaction centers and light saturation due to diffusion limitations, such as lower mesophyll conductance [42,48]. Inoculation with SL and SF significantly improved photochemical quenching under HM stress conditions, suggesting that these ECMF species enhance the resistance of poplar cuttings to Pb stress and improve the efficiency of PSII photochemistry. Under moderate HM stress conditions, cuttings inoculated with SL and SF exhibited higher nonphotochemical quenching than non-inoculated cuttings, indicating that mycorrhizal cuttings can protect plants from HM toxicity. The maximum quantum yield of photosystem II is a reliable diagnostic indicator of damage caused by environmental stresses [49]. Our results indicated that the maximum quantum yield of photosystem II in mycorrhizal plants inoculated with SL and SF under severe heavy metal stress was significantly higher than that seen in non-mycorrhizal plants. Additionally, the maximum quantum yield of photosystem II in all plants decreased under severe heavy metal stress, but the decrease in plants with SL and SF symbiosis was less pronounced than that seen in non-mycorrhizal plants.

Pb ions are the primary toxic ions for most plants in mining areas, and the accumulation of higher Pb levels in plants is essential for their phytoremediation potential [1,4]. Previous studies have reported that many plants, including *Sophora vivifolia* [1], *Pinus sylvestris* [33], and *Bidens parviflora* [4], exhibit higher Pb levels under HM stress. In our study, Pb levels in the shoots of cuttings inoculated with SL and SF were significantly lower than those in the roots. Notably, poplar cuttings inoculated with SL and SF accumulated higher levels of Pb ions than non-inoculated plants, particularly in the roots, which was consistent with the findings of previous studies [14,33]. Heavy metal stress increased the translocation factor in cuttings; however, cuttings inoculated with SL and SF exhibited a lower translocation factor than non-mycorrhizal cuttings under heavy metal stress. Therefore, ECMF inoculation did not inhibit the absorption of heavy metals by host plants; rather, it facilitated the transport of heavy metals into the rhizosphere and their absorption into plant roots through extensive hyphae [30,31]. When mycorrhizal plants grow better than their non-mycorrhizal counterparts despite accumulating more heavy metals, the beneficial effects of these microorganisms likely depend on molecular processes that alleviate stress [1,9,50].

In plants exposed to high concentrations of heavy metals, metal-binding proteins known as MTs are upregulated. These MTs play a crucial role in sequestering heavy metals and promoting optimal plant growth [10,13]. Both non-mycorrhizal and mycorrhizal plants exhibited similar degrees of upregulation of MT gene expression under HM stress conditions, suggesting that the plants may have adapted to metal-polluted soil, with persistent effects primarily attributed to fungal colonization. The enhanced tolerance of mycorrhizal cuttings inoculated with SL and SF was generally associated with higher MT gene expression in the roots. This suggests that these polypeptides may provide protection against HM-induced stress and stimulate the production of more HM-binding proteins for Pb ions [13]. Therefore, mycorrhizal cuttings that preferentially accumulate Pb ions in their roots generally exhibit higher tolerance, as their water status and photosynthetic capacity are protected from toxicity. Additionally, MT genes in ectomycorrhizal plants may also be involved in the tolerance process and require further investigation.

## 5. Conclusions

Our findings indicate that *P*. *alba* cuttings inoculated with SF exhibit greater phytoremediation potential regarding tolerance to Pb stress. We also observed that the positive effect of SL symbiosis was most pronounced under severe Pb stress, while the effects of GG and SV were not significant under Pb stress. Based on these results, SL and SF are suggested as potential species for ecological restoration in mining areas. These ECMF species can enhance phytoremediation efforts by improving plant tolerance to Pb stress and facilitating heavy metal accumulation. Overall, these findings offer valuable insights into the selection of suitable ECMF species for the effective phytoremediation of Pb-contaminated soils, with implications for ecological restoration in mining areas.

## Figures and Tables

**Table 1:** Gene primers used for quantitative real-time PCR amplification.

Gene Name	Primer-Forward (5′-3′)	Primer-Reverse (5′-3′)
*PaMT1*	ATGTCTGGCTGTAGCTGTGG	ACCATGTCCATGTGTCCTCAT
*PaMT2*	ATGCT TGCTGTGGTGGAAGC	GAATCAACGCAGCCAGC
*PaMT3*	ATGTCTAGCACCTGCGACAA	ACACATGACGGTTTACGTG
*UBQ* [10]	GCCCAGAGGTCCTCTTCCAA	GGGGCTAGTGCTGAGATTT

**Table 2:** ECMF colonization rates of *P. alba* under Pb stress.

Treatments		Colonization Rate (%)
Inoculation	Pb (mg kg^−1^)	
NM	0	0.00 ± 0.00 g
	200	0.00 ± 0.00 g
	400	0.00 ± 0.00 g
SL	0	33.12 ± 3.19 cd
	200	32.57 ± 3.89 d
	400	31.22 ± 2.38 d
SF	0	39.75 ± 2.63 a
	200	36.71 ± 3.81 b
	400	35.91 ± 2.22 bc
SV	0	15.95 ± 2.77 f
	200	15.80 ± 2.31 f
	400	14.55 ± 1.50 f
GG	0	25.39 ± 2.52 e
	200	22.63 ± 3.18 e
	400	17.13 ± 2.10 f

Note: different letters indicate significant difference at *p* ≤ 0.05; the data are means ± SD (n = 6).

**Table 3:** The effects of ECMF species and Pb stress on *P. alba* for all indexes.

Measurements	ECMF Species		Pb Treatment		ECMF Species × Pb Treatment	
	*F*	*p*	*F*	*p*	*F*	*p*
Height	46.23	0.00 **	28.46	0.00 **	1.64	0.13 ^NS^
Biomass of shoots	46.13	0.00 **	9.22	0.00 **	5.06	0.00 **
Biomass of roots	231.45	0.00 **	57.27	0.00 **	7.45	0.00 **
Root/Shoot	85.80	0.00 **	21.43	0.00 **	10.61	0.00 **
RWC	124.91	0.00 **	76.19	0.00 **	0.87	0.55 ^NS^
REL	20.82	0.00 **	60.87	0.00 **	3.61	0.00 **
Pn	51.02	0.00 **	55.85	0.00 **	4.95	0.00 **
Gs	33.21	0.00 **	113.45	0.00 **	9.33	0.00 **
Ci	2.99	0.024 *	51.60	0.00 **	2.18	0.038*
E	30.48	0.00 **	107.22	0.00 **	8.038	0.00 **
NPQ	6.91	0.00 **	10.03	0.00 **	1.41	0.21 ^NS^
qP	4.52	0.00 **	21.09	0.00 **	5.29	0.00 **
Fv/Fm	6.72	0.00 **	52.71	0.00 **	5.11	0.00 **
ΦPSII	3.19	0.018 *	17.62	0.00 **	1.53	0.16 ^NS^
Pb content in shoots	201.747	0.00 **	2909.69	0.00 **	68.43	0.00 **
Pb content in roots	13.71	0.00 **	2002.68	0.00 **	4.84	0.00 **
The translocation factor	112.24	0.00 **	1063.71	0.00 **	30.63	0.00 **
*PaMT1* expression in leaves	15.14	0.00 **	376.62	0.00 **	4.36	0.00 **
*PaMT2* expression in leaves	5.84	0.00 **	110.02	0.00 **	1.64	0.13 ^NS^
*PaMT3* expression in leaves	1.35	0.26 ^NS^	383.63	0.00 **	2.77	0.01 *
*PaMT1* expression in roots	11.08	0.00 **	868.89	0.00 **	3.65	0.00 **
*PaMT2* expression in roots	14.48	0.00 **	342.32	0.00 **	2.76	0.01 *
*PaMT3* expression in roots	5.12	0.00 **	230.91	0.00 **	4.26	0.00 **

Note: **: significant effect at *p* ≤ 0.01; *: significant effect at 0.01 < *p* ≤ 0.05; ^NS^: no significant effect.

## Data Availability

The original contributions presented in this study are included in the article. Further inquiries can be directed to the corresponding author.

## Abbreviations

ECMF, ectomycorrhizal fungi; ECM, ectomycorrhizal; HM, heavy metal; MT, metallothionein; SL, *Suillus luteus*; SF, *S*. *flavidus*; SV, *S*. *variegatus*; GG, *Gomphidius glutinosus*; RWC, relative water content; REL, relative electrolyte leakage; FW, fresh weight; TW, turgid weight; DW, dry weight; EC1, initial electrical conductivity; EC2, final electrical conductivity; Pn, net photosynthesis rates; Gs, stomatal conductance; Ci, intercellular CO_2_ concentration; E, transpiration rates; Fm, maximal fluorescence; Fo, minimum fluorescence; Fv/Fm, maximum quantum yield; ΦPSII, actual quantum yield; qP, photochemical quenching; NPQ, nonphotochemical quenching; TF, translocation factor; UBQ, ubiquitin gene; ANOVAs, analyses of variance; PCA, principal component analysis; NM, non-inoculation.
